# Taxonomic Description of *Stenodiplosis tectori* n. sp. (Diptera: Cecidomyiidae), a Seed Parasite of Cheatgrass, *Anisantha tectorum*, Based on Morphological and Mitochondrial DNA Data

**DOI:** 10.3390/insects12080755

**Published:** 2021-08-22

**Authors:** Brian G. Rector, Raymond J. Gagné, Juan Manuel Perilla López, Kirk C. Tonkel, Marie-Claude Bon, Fatiha Guermache, Massimo Cristofaro

**Affiliations:** 1 Great Basin Rangelands Research Unit, USDA-ARS, 920 Valley Road, Reno, NV 89512, USA; kirk.tonkel@usda.gov; 2 Systematic Entomology Laboratory (Emeritus), USDA-ARS, c/o Smithsonian Institution, MRC-168, P.O. Box 37012, Washington, DC 20013, USA; rgagne@rcn.com; 3 Department of Biological Sciences, Wright State University, 3640 Colonel Glenn Highway, Dayton, OH 45435, USA; jmperillal@gmail.com; 4 European Biological Control Laboratory, Campus International de Baillarguet, USDA-ARS, 34980 Montferrier-sur-Lez, France; mcbon@ars-ebcl.org (M.-C.B.); fguermache@ars-ebcl.org (F.G.); 5 Biotechnology and Biological Control Agency, via Angelo Signorelli 105, 00123 Rome, Italy; m.cristofaro55@gmail.com; 6 ENEA C.R. Casaccia, SSPT-BIOAG-PROBIO, via Anguillarese 301, 00123 Rome, Italy

**Keywords:** annual grass, *Bromus* sensu lato, classical biological control of weeds, invasive species, Poaceae, wildfire

## Abstract

**Simple Summary:**

Cheatgrass is an annual grass species from Eurasia that has become invasive in much of western North America. It has been implicated in the recent increases in the frequency, size, and intensity of wildfires, contributing to severe economic, environmental, and social destruction. In order to reduce this damage, the USDA-ARS established a classical biological control program against cheatgrass. In 2018 and 2019, adult gall midges were collected emerging from cheatgrass seed heads collected at several sites in Bulgaria and Greece; this is the first gall midge ever recorded from cheatgrass. Morphological and DNA barcode comparisons with related midge species recorded from other plant hosts revealed that this midge from cheatgrass is a new species, described here as *Stenodiplosis tectori* n. sp. The present study is the first to report DNA barcode data in the genus *Stenodiplosis*. The DNA barcode results indicated relatively high year-to-year within-population diversity. Implications for this gall midge’s utility as a biological control agent of cheatgrass are discussed.

**Abstract:**

Cheatgrass is an annual grass species from Eurasia that has become invasive in much of western North America. It has been implicated in recent increases in the frequency, size, and intensity of wildfires, contributing to severe economic, environmental, and social destruction. In order to reduce this damage, the USDA-ARS established a classical biological control program against cheatgrass. In 2018 and 2019, adult gall midges were collected emerging from cheatgrass seed heads collected at several sites in Bulgaria and Greece; this is the first gall midge ever recorded from cheatgrass. Morphological comparisons with related midge species recorded from other plant hosts revealed that this midge from cheatgrass is a new species, described here as *Stenodiplosis tectori* n. sp. This status was supported by sequence comparisons of a barcode region of the gene encoding the mitochondrial cytochrome *c* subunit I (*CO1*) protein in *Stenodiplosis tectori* n. sp. and three congeners. The present study is the first to report MT-CO1 data in the genus *Stenodiplosis*. The ingroup *Stenodiplosis tectori* n. sp. collected in the Balkans grouped in one phylogenetic supported clade, with an average K2P-distance from its closest related congener, *S. sorghicola*, of 7.73% (SD = 1.10). The findings indicated relatively high year-to-year within-population diversity. Implications for this gall midge’s utility as a biological control agent of cheatgrass are discussed.

## 1. Introduction

Cheatgrass, a.k.a. downy brome, *Anisantha* (= *Bromus*) *tectorum* (L.) Nevski, is a winter annual grass native to Eurasia that has become invasive on millions of hectares in western North America [1,2,3]. In its invaded range, senesced cheatgrass plants dry and remain in place without disintegrating, providing fine fuel that ignites readily and can carry fire to larger fuel sources (e.g., shrubs, conifers), thereby contributing to increases in frequency, size, and intensity of wildfires in the western USA in recent decades [4,5]. A cheatgrass-fire cycle has been observed in which cheatgrass promotes fire by altering the fuel bed [4] and fire appears to promote further cheatgrass invasion via altered resource availability [6,7] and increased seed dispersal [8]. Remarkably, the Old World species cheatgrass has also formed a symbiotic association in its invaded range with a New World fungal endophyte that allows its seeds to better withstand heat from fire, thus increasing the invasive and destructive potential of this cheatgrass-endophyte meta-organism [9].

Expansive portions of the sagebrush steppe, a unique and endangered ecosystem, have been converted into cheatgrass monocultures as cheatgrass-dominated landscapes have burned 2–4 times as frequently as native Great Basin vegetation types since 1980 [5]. California and the Intermountain West have recently experienced large-scale catastrophic fires and replacement of native sagebrush-dominated landscapes with invasive annual grasses, reinforced by additional wildfires; this will be difficult to reverse [10]. Annual grass monocultures and associated depletion of soil moisture and nutrients, reduced forage production, plant diversity, and community productivity; and acceleration of wildfire cycles [4,5,10,11,12,13] present an urgent challenge for land managers and landowners in the western USA. Due to the scale of the cheatgrass invasion, the rockiness of the soils, and steep terrain, the use of herbicides or other high-input management strategies are limited to the highest-priority parcels. In addition, attempts to reduce cheatgrass impacts through reseeding have occurred for >70 years [14] with little sustainable success. As such, a classical biological control program was established by the USDA-ARS to discover co-evolved natural enemies of cheatgrass and evaluate them for their suitability as potential biological control agents that would reduce cheatgrass populations to less economically, environmentally, and socially destructive levels [15] (USDA-ARS Research Project #439311). This program has focused on exploration of the native Eurasian range of cheatgrass to better understand the natural history of this plant and to discover herbivorous arthropods, such as mites, gall midges, and other small insects with life cycles rapid enough to match that of cheatgrass.

**Figure 1:**
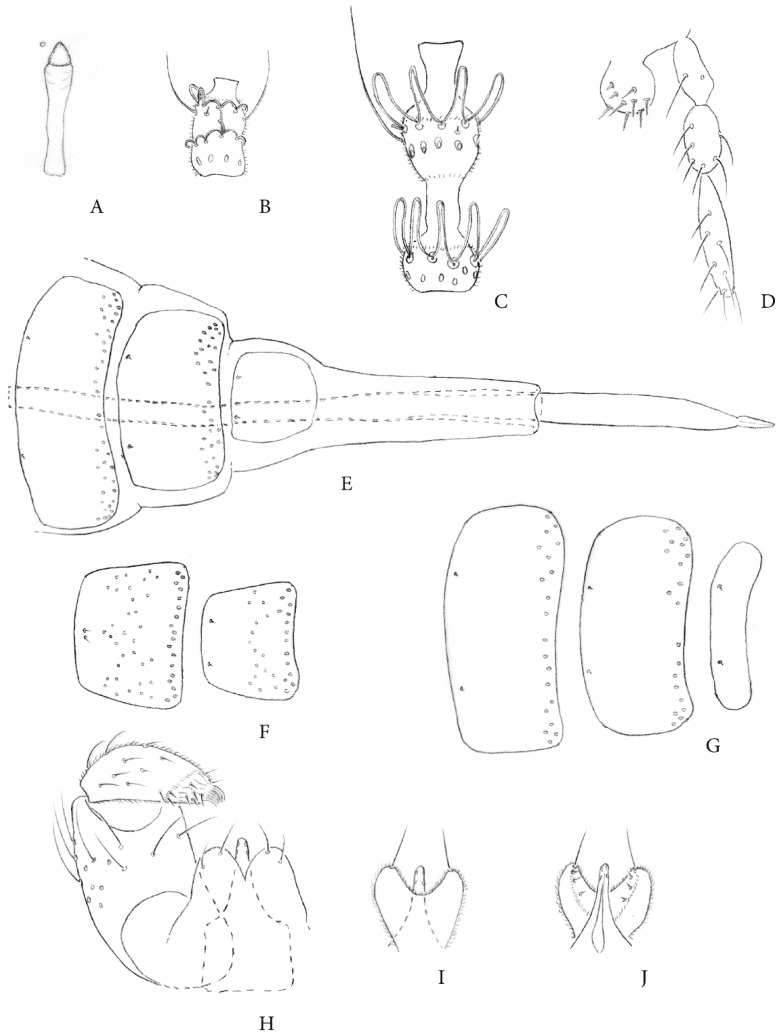
*Stenodiplosis tectori.* (**A**), Third instar spatula and associated papillae. (**B**), Female fourth flagellomere (lateral). (**C**), Male fourth flagellomere (dorsal). (**D**), Labellum and palpus. (**E**), Female abdomen, sixth segment to cerci (dorsal). (**F**), Male seventh and eighth sternites. (**G**), Male sixth to eighth tergites. (**H**), Male terminalia, one gonopod removed (dorsal). (**I**), Hypoproct and aedeagus (dorsal). (**J**), Same (ventral).

In the course of exploration for potential biocontrol agents of cheatgrass, several dozen specimens of a gall midge (Diptera: Cecidomyiidae) were discovered from mature cheatgrass spikes in northeastern Greece and identified to the genus *Stenodiplosis* Reuter. *Stenodiplosis* is a cosmopolitan genus of 12 species of monophagous or closely oligophagous gall midges that feed in grass seeds [16]. These include the sorghum midge, *Stenodiplosis sorghicola* (Coquillett), a pest of sorghum wherever that plant is grown. The genus is assigned to the subfamily Cecidomyiinae and the tribe Cecidomyiini [17]. Adults share the following characters: male flagellomeres have two similarly shaped spherical nodes, each with a looped circumfilum, the nodes separated by a cylindrical internode, and all but the last flagellomere terminating in a cylindrical neck (Figure 1C); tarsal claws are simple and curved beyond midlength; abdominal tergites have a pair of anterior trichoid sensilla and lack lateral setae (Figure 1G); and the ovipositor is greatly attenuated and gradually tapered to the tiny, narrow, dorsoventrally flattened and closely juxtaposed cerci (Figure 1E). In adults, only the loss of lateral setae on the abdominal tergites separates this genus from its closest relative *Contarinia* Rondani [17]. The larvae of *Stenodiplosis*, however, are much more distinctive among Cecidomyiini for their ovoid shape and caudally situated hind abdominal spiracles (Figure 15 in [18]) and the lack of apparent setae on all papillae except on two of each triplet of lateral papillae.

The goal of this study was to determine whether gall midge specimens collected from cheatgrass populations in southeastern Europe represented a new species, using morphological and molecular data. Herein, we formally describe a new species, *Stenodiplosis tectori* n. sp., a new candidate for biological control of cheatgrass.

## 2. Materials and Methods

### 2.1. Field Collections of Specimens

The gall midge specimens were collected by digging up mature cheatgrass plants with spikes, including roots and placing them in perforated zip-top plastic bags with moistened paper towels to maintain turgidity in the plants. Plants in bags were maintained in a cool, dark, well-ventilated space until imagos of the gall midges emerged from the cheatgrass spikes. Afterwards, cheatgrass florets were dissected to check for evacuated puparia. Plants infested with gall midges were collected near Maroneia, Greece in 2018 and 2019; additional collections of gall midges occurred at Kalabaka, Greece and Hisarya, Bulgaria in 2019 (see Table 1). Three related species collected near Brookings, SD, USA, in 2015 provided specimens for molecular comparisons: *Stenodiplosis bromicola* Marikovskij and Agafonova, from smooth brome, *Bromopsis* (= *Bromus*) *inermis* (Leyss.) Holub, a relative of cheatgrass; *Stenodiplosis geniculati* Reuter, from *Alopecurus arundinaceus* Poir.; and *Stenodiplosis sorghicola* Coquilett from *Andropogon gerardii* Vitman.

### 2.2. Taxonomic Studies

Specimens collected in Maroneia, Greece in 2018 were cleared for taxonomical study in KOH and mounted in Canada balsam using techniques outlined in Gagné 1989 [19]; a separate series of specimens from the same collections was mounted in Keiferʹs F medium following clearing in lactic acid for 5 to 10 d at 30 °C. The latter series was adequate for identification but not for detailed scrutiny, as musculature that remained obscured some features. A glossary of adult morphological terms can be found in Gagné 2018 [17]. Larval anatomy follows Gagné 1989 [19]. All lengths are given in mm. Scanning electron micrographs of female cerci were made using a Zeiss EVO MA 15. Line drawings were made by RJG with the use of a camera lucida attached to a Wild phase contrast microscope. Typically, most of the scales and setae become lost in the mounting process, but enough remain to show that the setae leave larger sockets than do scales. The illustrations show these sockets in their actual placement and number and any setae and scales are drawn to their approximate actual length and thickness. The holotype and paratypes of the new species are deposited in the insect collection of the National Museum of Natural History in Washington, DC (USNM).

### 2.3. Molecular and Phylogenetic Analyses

Adult gall midges collected in Maroneia, Greece in 2018 (ST-01; Table 1) that were morphologically identified by RJG as *S. tectori* n. sp. were shipped to JMPL at WSU in 96% EtOH for molecular analyses. Genomic DNA (gDNA) was performed on a whole, destructively sampled specimen using the DNeasy Blood and Tissue Kit (Qiagen, Valencia, CA, USA). The barcode region of the gene encoding subunit I of the mitochondrial cytochrome c oxidase (MT-CO1) protein was amplified by polymerase chain reaction (PCR), using the universal Folmer primers [20]. PCR amplifications run at WSU were run on a Mastercycler pro thermocycler (Eppendorf, Enfield, CT) using the following thermal conditions: 2 min at 94 °C; four cycles of 94 °C for 30 s, 50 °C for 30 s, and 72 °C for 60 s; six cycles of 94 °C for 30 s, 48 °C for 30 s, and 72 °C for 60 s; 36 cycles of 94 °C for 30 s, 45 °C for 30 s, and 72 °C for 60 s; and a final extension at 72 °C for 7 min. MT-CO1 fragments were obtained in the same manner from additional adult specimens of *S. bromicola*, *S. sorghicola*, and *S. geniculati*; all collected in Brookings, SD in 2015 (Table 1). One MT-CO1 product for each species was sent to the University of Arizona Genomics Core, Tucson, AZ, USA, for sequencing in the forward and reverse directions.

Gall midge adults collected in 2019 in Maroneia and Kalabaka, Greece and Hisarya, Bulgaria were dissected to separate the abdomens from the rest of the body. The abdomens were sent to RJG for morphological identification (to ensure they belonged to the same species as the holotype described below) while the heads and thoraces were shipped to the USDA-ARS European Biological Control Laboratory, Montpellier, France for molecular analyses. Genomic DNA of the head and thorax of each specimen was non-destructively extracted using a modified protocol for the DNeasy Blood and Tissue Kit (Qiagen, Hilden, Germany) [21]. The MT-CO1 fragment was amplified from each sample by PCR in a 30 μL reaction volume including one active unit of Qiagen *Taq* DNA Polymerase (Qiagen, Hilden, Germany), 1× CoralLoad Qiagen PCR Buffer (1.5 mM MgCl_2_), 0.2 mM of each dNTP, 0.2 mg/mL of bovine serum albumin (BSA), 0.2 μM of the Folmer primers [20], and 2 μL template DNA. All PCR amplifications were run on a 9700 thermocycler (Applied Biosystem ^®^) using the following thermal conditions: an initial denaturation of 94 °C for 3 min; followed by 40 cycles of denaturation for 30 s at 94 °C, annealing for 1 min at 50 °C, and elongation of 1 min at 72 °C; then a final elongation step of 10 min at 72 °C. The PCR products were sequenced in the forward and reverse directions using the Sanger approach by Genoscreen, Lille, France.

All MT-CO1 sequences obtained in this study were translated into amino acids; the absence of stop codons was checked with CodonCode Aligner (v.8) (CodonCode Corp., Centerville, MA). Species matching using these sequences was attempted via the BOLD identification engine in Barcode of Life Data Systems and via BLAST queries of the National Center for Biotechnology (NCBI) database. All MT-CO1 sequences were aligned using MUSCLE as implemented in MEGA X [22]. The dataset was cut to the length of the shortest sequences (Table 1), giving a final alignment of 17 sequences of 615 nucleotides each, including *S. tectori* n. sp. (13), *S. bromicola* (1), *S. sorghicola* (1), *S. geniculati* (1) and one sequence from the contribal species *Contarinia jongi* (MW582602.1), to serve as an outgroup taxon. Basic sequence data, such as the nucleotide composition and the number of variable sites and parsimony informative sites were taken from MEGA X. Pairwise distance calculations between all MT-COI sequences were computed using Kimura’s 2-parameter (K2P) distance model [23] for all codon positions through MEGA X. A Bayesian approach (BA) was employed to reconstruct phylogenetic relationships between taxa as implemented in MrBayes [24]. The most appropriate model of evolution to be used in BA, which was inferred by MODELTEST as implemented in MEGA X, was T92+G using the Bayesian Information Criterion. Two simultaneous runs of 1 million generations were performed and convergence was maximized by ensuring that the average standard deviation of split frequencies fell below 0.01 and potential scale reduction factors approached 1.0. The first 25% of each run was discarded as burn-in phase for the estimation of the consensus topology and the computation of the posterior probability for each node.

## 3. Results

### 3.1. Field Collections of Specimens

*Stenodiplosis* adults were reared out from mature cheatgrass spikes collected in the spring of 2018 near the town of Maroneia, Greece (40.87°, 25.53°). Further surveys in the spring of 2019 recovered additional specimens from Maroneia, as well as from cheatgrass populations near Kalabaka, Greece (39.705°, 21.612°) and Hisarya, Bulgaria (42.515°, 24.727°). Adult midges emerged from cheatgrass florets and some evacuated puparia were found within the florets. Adult male and female specimens were examined and determined to be a new species of *Stenodiplosis*.

### 3.2. Taxonomic Description

*Stenodiplosis tectori* Gagné and Perilla, new species.

**Figure 2:**
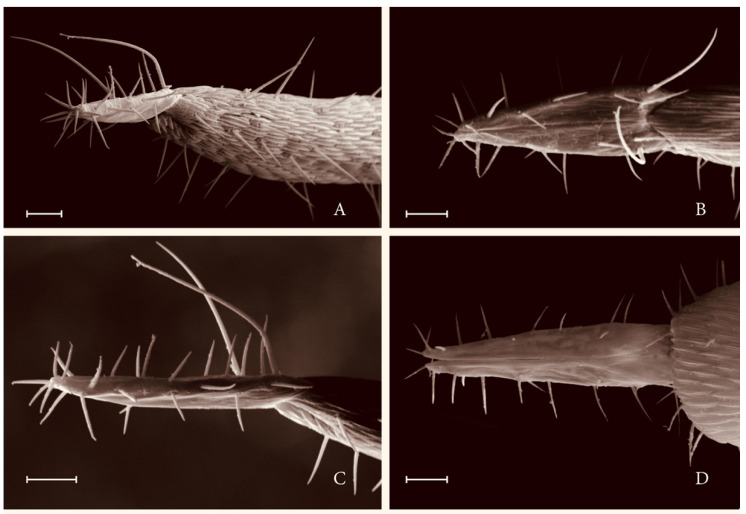
*Stenodiplosis tectori*, female cerci and part of adjoining protrusible ovipositor. (**A**), Caudolateral view. (**B**), Dorsal view (not flat). (**C**), Lateral view. (**D**), Ventral view. Scale bar = 10 µm.

Figure 1 and Figure 2.

Adult. Head: Eyes connate, 3–4 facets long at vertex, facets circular, slightly farther apart laterodorsally than elsewhere. Frons with 11–15 setae. Mouthparts (Figure 1D): labella spheroid, setulose, each with several thick setae; palpus with three segments—the first two ovoid, the last elongate, linear. Male antennal flagellomeres (Figure 1C) with internode and necks approximately as long as first node. Female flagellomeres (Figure 1B) cylindrical, with short necks, some circumfilar loops bowed away from dorsum of flagellomere.

Thorax: Wing hyaline, length in male, 2.3–2.5 (n = 10), in female, 2.2–2.4 (n = 10); R_5_ with slight curve, joining C at wing apex; Rs evanescent; wing fold barely evident; M_4_ and CuA forming a fork. Acropods approximately 1/3 length of fifth tarsomeres; claws untoothed, curved beyond midlength; empodia slightly longer than claws; pulvilli ca. one-third the length of empodia.

Male abdomen (Figure 1F–J): Tergites rectangular, all lacking scales, first through fourth with posterior setae in a mostly single, medially discontinuous row, fifth through seventh with mostly double row of posterior setae nearly or wholly continuous medially, all with anterior pair of trichoid sensilla, eighth apparent only on anterior third, without vestiture except for anterior pair of trichoid sensilla. Sternites second through seventh quadrate with mostly single row of posterior setae, setae and setiform scales on posterior two-thirds, and with anterior pair of adjacent trichoid sensilla; eighth sternite similar to preceding but with less distance between posterior and more anterior field of setae, the pair of trichoid sensilla well separated.

Terminalia: cercus ellipsoid, with a few setae along distal edge; hypoproct thick, deeply notched and concave medially, the resulting cylindrical lobes broadly rounded apically, each with a long apical seta and several short setae medially; gonocoxite short-cylindrical; gonostylus broadest near base, setulose except glabrous and ridged dorso-apically, with several short, scattered setae throughout; apodeme broad with right-angled anterior margin; aedeagus tapered, approximately as long as hypoproct, with pair of sensilla on each side.

Female abdomen (Figure 1E and Figure 2A–D): Tergites first through seventh as in male, eighth tergite quadrate, approximately half width of seventh, without setae except for pair of anterior trichoid sensilla. Sternites as for male except seventh sternite without space between posterior and more anterior field of setae and eighth sternite evanescent, marked only by presence of anterior pair of widely separated trichoid sensilla. Protrusible part of ovipositor approximately 7.5 times as long as seventh tergite. Cerci elongate, gradually tapered, asetulose, with a pair of elongate basal setae approaching three-quarters the length of cerci, the remaining setae all of nearly uniform length, two subapical pairs slightly thicker than remainder.

Third instar larva. Spatula (Figure 1A) with triangular anterior tooth, shaft long, parallel-sided, weakly pigmented. Papillae in full complement as described by Möhn 1955 [25] for the supertribe Cecidomyiidi. As with the type species, *Stenodiplosis geniculati* Reuter, all papillae devoid of setae except miniscule seta present on two of each triplet of lateral setae [19].

HOLOTYPE, male, Maroneia, Greece, 40.87°, 25.53°, 30-IV-2018, emerged 2-V-2018, B. G. Rector, deposited in USNM. PARATYPES, 7 males, 11 females, 3 larvae, same data as holotype. Other specimens examined: 6 females on SEM stubs, same data as holotype.

#### 3.2.1. Etymology

The species name is the genitive singular of *tectorum*, the specific epithet of the host plant.

#### 3.2.2. Notes on Differential Diagnosis

*Stenodiplosis tectori* was compared to types or authoritatively identified specimens of nine of the 12 previously described species [17] represented in the USNM collection and to published descriptions of the three remaining species, which are *Stenodiplosis panici* Plotnikov, *Stenodiplosis dactylidis* Barnes, and *Stenodiplosis stenotaphri* Barnes. The new species is distinct and readily separated from all 12 congeners by the presence in the larva of a spatula, in the female of an elongate pair of basal cercal setae that are at least ¾ the length of the cerci, and in the male by the dorsal non-setulose, ridged area basad of the tooth. This last character state is not found in any of the Nearctic or Eurasian species. Only the three African species, *S. sorghicola* (Coquillett) (now cosmopolitan as noted above), *Stenodiplosis sorghi* Harris and *Stenodiplosis gambae* Harris, have the male cerci partially glabrous and ridged but in those species the area extends over almost the entire dorsal surface. A further difference between the new species and the other 12 is that the acropods are only approximately one-third the length of the fifth tarsomeres. The nine other *Stenodiplosis* spp., for which specimens were examined have acropods that are more than half the length of the fifth tarsomeres. This character can still serve to separate most *Stenodiplosis* spp., from *Contarinia* but is no longer exclusive. Before the discovery of the present species, a character shared by all *Stenodiplosis* spp., was the lack of a larval spatula. One is present in the new species although reduced in size (Figure 1A) compared to the typical clove-shaped spatula found in *Contarinia*.

### 3.3. Molecular and Phylogenetic Analyses

Barcode compliant sequences of 615 nt were produced from *S. tectori* n. sp. specimen ST-01 and the three congeners, while longer sequences of 657 nt in length were generated from all other *S. tectori* n. sp. specimens collected in 2019 and analyzed; all sequences have been deposited in GenBank with accession numbers indicated in Table 1. In BOLD and BLAST queries, all sequences could be assigned only to the family level of Cecidomyiidae with a similarity score ranging from 91% to 100%. To our knowledge, the sequences obtained in the present study are the first ones generated for *Stenodiplosis*.

**Figure 3:**
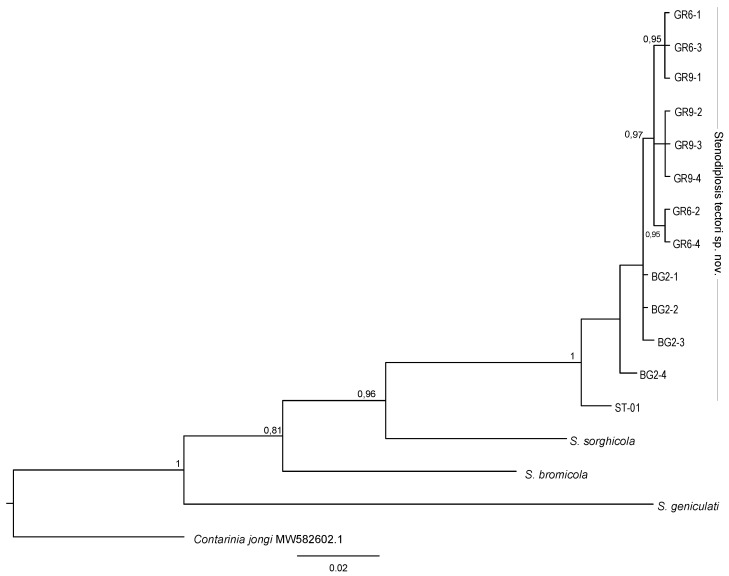
Bayesian phylogenetic tree of *S. tectori* MT-CO1 sequences, three congeners, and one contribal outgroup. Only significant posterior probabilities (>0.8) obtained from the Bayesian analysis are shown at the nodes. The scale bar indicates the expected number of substitutions per site. Tips labels refer to specimen IDs listed in Table 1.

A total of seven haplotypes were recovered from the 13 *S. tectori* n. sp. specimens analyzed, six of which (85.7%) were “private” haplotypes, i.e., unique to a single collection; one of the most common haplotypes, H5, was shared between the two localities in Greece (Table 1). The final alignment for species delimitation including the outgroup included 130 variable nucleotides out of 615, of which 61 were parsimony informative. The pairwise K2P distances within *S. tectori* averaged 0.0067 ± 0.0017 whereas the pairwise distances between *S. tectori* n. sp. and its closest relative *S. sorghicola* was 0.0773 ± 0.011, and the pairwise distance between its three congeners averaged 0.1163 ± 0.0116 (Table 2). The tree topology obtained by BA is illustrated in Figure 3. Bayesian analysis showed that MT-COI sequences from *S. tectori* specimens collected in the Balkans grouped in one supported clade, while forming no sister groups with the other three species.

## 4. Discussion

Phylogenetic analysis (Figure 3) showed a strongly supported *S. tectori* n. sp. ingroup, distinct from its congeners. Although our study is based on a relatively small number of specimens that could be sequenced, in particular from 2018, it resulted in the discovery of remarkable genetic variation within *S. tectori* n. sp., particularly between specimens collected in different years (2018 and 2019) at the same location (Maroneia, Greece). The haplotype H1 (collected at Maroneia in 2018) was more closely related to the haplotype H4, from Hisarya, Bulgaria, and to a lesser extent to H2 and H3, than to haplotypes H5 and H6 from the same site but collected one year later in 2019, the latter of which were relatively close to each other, as well as to H7 collected in Kalabaka, Greece in 2019. One possible explanation is inherent to sampling bias and that H1 co-occurred with H5 and H6 in 2019 but was not sampled. These results suggest that efforts to investigate this midge in Bulgaria and Greece should be intensified in order to make conclusive intraspecific diversity assessments using MT-CO1 sequence data, as well as broader genetic comparisons (e.g., genotyping by sequencing). Another possible explanation for this result is that the populations that produced H1 and H4 are not reproductively isolated, despite being separated by almost 200 km, “as the gall midge flies.” While this distance is not trivial, it is not inconceivable that strong winds may move adult gall midges great distances, resulting in sporadic genetic admixture and heterogeneous populations that would benefit in theory from reduced inbreeding. In the context of potential future releases of *S. tectori* n. sp. in the USA for biological control of cheatgrass, this result underscores the importance of genetically characterizing colonies that are petitioned for release following host specificity and other biological evaluations. It must not be assumed that gall midges taken from the same site are all genetically identical, particularly when they are collected in different years.

It is also notable in the context of classical biological control that as a new species to science, *S. tectori* n. sp. has never been described from any other host plant before, not least from any economically important grass species. This is significant, given the importance of host specificity and avoidance of non-target attack by candidate biocontrol agents. Judging from the extensively studied biology of its close relative, the seed parasite *S. bromicola* [26,27], and observations of evacuated puparia of *S. tectori* n. sp. within the florets of infested cheatgrass plants, where one would otherwise expect to find seeds, it can be hypothesized that if *S. tectori* n. sp. were to attack economically important cereals, such as wheat, oats, or barley, its damage to these plants would be quite conspicuous and it would already be well known as a pest, just as *S. bromicola* is a known pest of the forage grass, smooth brome. Thus, the status of *S. tectori* n. sp. as a new species suggests a relatively high host specificity and lack of threat to economically important plant species.

## Figures and Tables

**Table 1:** List of the sequenced specimens used in this study with respective collection records and associated GenBank accession numbers. Grey background with n/a = not applicable.

*S. tectori* Specimen ID/*Stenodiplosis* Species	Locality	Collector	Collection Date (dd/mm/yyyy)	Latitude, Longitude	MT-CO1 Fragment Length (bp)	*S. tectori* Haplotype	GenBank Accession Number
ST-01	Maroneia, Greece	B.G.R.	30/04/2018	40.87°, 25.53°	615	H1	MZ382457
BG2-1	Hisarya, Bulgaria	M.C.	01/06/2019	42.515°, 24.727°	657	H2	MZ382458
BG2-2	Hisarya, Bulgaria	M.C.	01/06/2019	42.515°, 24.727°	657	H2	MZ382459
BG2-3	Hisarya, Bulgaria	M.C.	01/06/2019	42.515°, 24.727°	657	H3	MZ382460
BG2-4	Hisarya, Bulgaria	M.C.	01/06/2019	42.515°, 24.727°	657	H4	MZ382461
GR6-1	Maroneia, Greece	M.C.	15/05/2019	40.87°, 25.53°	657	H5	MZ382462
GR6-2	Maroneia, Greece	M.C.	15/05/2019	40.87°, 25.53°	657	H6	MZ382463
GR6-3	Maroneia, Greece	M.C.	15/05/2019	40.87°, 25.53°	657	H5	MZ382464
GR6-4	Maroneia, Greece	M.C.	15/05/2019	40.87°, 25.53°	657	H6	MZ382465
GR9-1	Kalabaka, Greece	M.C.	16/05/2019	39.705°, 21.612°	657	H5	MZ382466
GR9-2	Kalabaka, Greece	M.C.	16/05/2019	39.705°, 21.612°	657	H7	MZ382467
GR9-3	Kalabaka, Greece	M.C.	16/05/2019	39.705°, 21.612°	657	H7	MZ382468
GR9-4	Kalabaka, Greece	M.C.	16/05/2019	39.705°, 21.612°	657	H7	MZ382469
*S. bromicola* ST-03	Brookings, SD, USA	J.M.P.L.	05/06/2015	44.5°, −96.53°	615	n/a	MZ382470
*S. sorghicola* ST-04	Brookings, SD, USA	J.M.P.L.	05/06/2015	44.319°, −96.774°	615	n/a	MZ382471
*S. geniculati* ST-02	Brookings, SD, USA	J.M.P.L.	05/06/2015	44.5°, −96.53°	615	n/a	MZ382472

**Table 2:** Matrix of pairwise K2P distances within *Stenodiplosis tectori* n. sp. and between *Stenodiplosis tectori* n. sp. and the other three congeners (*S. bromicola*, *S. sorghicola* and *S. geniculati*). Data are expressed as the mean ± S.E. Grey background = not applicable.

	*S. tectori * (n = 13)	*S. sorghicola* (n = 1)	*S. geniculati* (n = 1)	*S. bromicola* (n = 1)
*S. tectori* (n = 13)	0.0067 ± 0.0017	0.0773 ± 0.011	0.129 ± 0.0145	0.0905 ± 0.0119
*S. sorghicola* (n = 1)			0.1295 ± 0.0149	0.0918 ± 0.0124
*S. geniculati* (n = 1)				0.1275 ± 0.0146

## Data Availability

The data presented in this study were generated within official duties of United States government personnel and are in the public domain. They are available upon request from the corresponding author.
